# LED Lighting – Modification of Growth, Metabolism, Yield and Flour Composition in Wheat by Spectral Quality and Intensity

**DOI:** 10.3389/fpls.2018.00605

**Published:** 2018-05-04

**Authors:** István Monostori, Márk Heilmann, Gábor Kocsy, Marianna Rakszegi, Mohamed Ahres, Susan B. Altenbach, Gabriella Szalai, Magda Pál, Dávid Toldi, Livia Simon-Sarkadi, Noémi Harnos, Gábor Galiba, Éva Darko

**Affiliations:** ^1^Agricultural Institute, Centre for Agricultural Research, Hungarian Academy of Sciences, Martonvásár, Hungary; ^2^Festetics Doctoral School, Georgikon Faculty, University of Pannonia, Keszthely, Hungary; ^3^Western Regional Research Center, United States Department of Agriculture-Agricultural Research Service, Albany, CA, United States; ^4^Department of Food Chemistry and Nutrition, Szent István University, Budapest, Hungary

**Keywords:** amino acid, indoor cultivation, glutathione, gluten, LED lighting, photosynthesis, wheat, yield quality

## Abstract

The use of light-emitting diode (LED) technology for plant cultivation under controlled environmental conditions can result in significant reductions in energy consumption. However, there is still a lack of detailed information on the lighting conditions required for optimal growth of different plant species and the effects of light intensity and spectral composition on plant metabolism and nutritional quality. In the present study, wheat plants were grown under six regimens designed to compare the effects of LED and conventional fluorescent lights on growth and development, leaf photosynthesis, thiol and amino acid metabolism as well as grain yield and flour quality of wheat. Benefits of LED light sources over fluorescent lighting were manifested in both yield and quality of wheat. Elevated light intensities made possible with LEDs increased photosynthetic activity, the number of tillers, biomass and yield. At lower light intensities, blue, green and far-red light operated antagonistically during the stem elongation period. High photosynthetic activity was achieved when at least 50% of red light was applied during cultivation. A high proportion of blue light prolonged the juvenile phase, while the shortest flowering time was achieved when the blue to red ratio was around one. Blue and far-red light affected the glutathione- and proline-dependent redox environment in leaves. LEDs, especially in Blue, Pink and Red Low Light (RedLL) regimens improved flour quality by modifying starch and protein content, dough strength and extensibility as demonstrated by the ratios of high to low molecular weight glutenins, ratios of glutenins to gliadins and gluten spread values. These results clearly show that LEDs are efficient for experimental wheat cultivation, and make it possible to optimize the growth conditions and to manipulate metabolism, yield and quality through modification of light quality and quantity.

## Introduction

Plant breeders frequently use indoor cultivation methods to accelerate their breeding projects independently of seasonal outdoor climatic conditions. However, growth chambers or greenhouses utilizing artificial light sources, such as fluorescent lamps or metal-halide lamps are often inefficient due to high operation temperatures, low efficiencies of light fluence and inadequate spectral distributions for optimal growth (Schettini, 2005; Morrow, 2008; Darko et al., 2014). High operation temperatures make it difficult to use fluorescent and metal-halide lamps close to the plants, thus the light intensity cannot be increased to optimal values. In addition, the energy consumption of these lamps is relatively high.

The utilization of Light-Emitting Diode (LED) technology can improve the efficiency of indoor plant cultivation (Singh et al., 2015). Combinations of different types of LEDs can provide high fluence and customized wavelengths for plant cultivation. At the same time, LEDs have low energy consumptions, long lifetimes and stable spectral distributions (Yeh and Chung, 2009). In spite of these advantages, the utilization of LEDs in phytotrons for plant cultivation is not prevalent yet. LED light systems are mainly used commercially for leafy plants, vegetables, fruits and horticultural plants to optimize plant productivity and quality year round.

The importance of light intensity and spectral distribution on plant growth and development is evident when plant cultivation is compared under different light environments. Through photosynthesis, light, especially blue and red wavelengths provides energy required for plant growth and development, but through photoreceptors, light regulates several morphogenetic processes including plant elongation, leaf expansion, stomatal opening, circadian clock and flowering (Chen et al., 2004). At the biochemical level, the light spectra influence both primary and secondary metabolism, affecting nutritional quality, carbohydrate and nitrogen metabolism, the production of flavor, color, volatile and aromatic compounds, as well as plant defense mechanisms (Colquhoun et al., 2013; Olle and Viršilė, 2013; Darko et al., 2014). For instance, red light decreased nitrate content, while sugar content and the amounts of tocopherols and phenolic compounds were increased in lettuce (Samuoliene et al., 2011, 2012). Blue light induced accumulation of anthocyanins and increased antioxidant capacity in lettuce (Li and Kubota, 2009). Moreover, it increased antioxidant activity and ascorbic acid content in cabbage and tobacco, contributing to protection against cucumber mosaic virus infection in tobacco (Li et al., 2012; Chen et al., 2015). In tomato, blue light enhanced antioxidant status and proline accumulation, suppressing the symptoms of gray mold infection (Kim et al., 2013). Franklin and Whitelam (2007) reported that the modified light quality was also able to attenuate injury caused by abiotic stressors, such as freezing temperatures. For example, the application of light with a decreased red/far-red ratio increased frost tolerance in Arabidopsis plants via increased *CBF* gene expression. A short far-red treatment increased the expression level of the CBF-regulon and increased frost tolerance in wheat and barley plants (Gierczik et al., 2017; Novák et al., 2017). These examples demonstrate that changes of light environment can affect several plant responses and metabolic processes. LEDs thus provide opportunities to manipulate the growth period, plant metabolisms, defense and the amount and quality of plant products (De Salvador et al., 2008; Schettini et al., 2011).

Wheat is one of the most important crops worldwide and huge research efforts are focused on studying physiological and molecular processes and stress responses. In many experiments, plants are grown under controlled environmental conditions, mainly in phytotrons that produce different, but mainly suboptimal light conditions in either intensity or spectral distribution for cereals.

Only a few investigations have examined the growth and development of cereals under LED lighting. In one of the early investigations, (Goins et al., 1997) reported that red LED alone is inadequate for wheat cultivation. Besides blue and red LEDs, the addition of far-red light resulted in taller plants and higher yield production in oat and barley (Lethin and Bankestad, 2016). Koga et al. (2013) demonstrated that blue and red LEDs affected the growth, *N*-metabolism and vitamin C and E contents of young barley plants when compared to natural light. Dong et al. (2014) studied sugar, protein and starch contents of wheat grain from plants produced under red, blue and white LEDs. However, they did not examine the breadmaking quality of the resulting flour.

In the present work, wheat plants were grown in plant growth chambers using different spectral distributions provided by LED light sources at intensities similar to those produced by fluorescence lamps. The effect of elevated light intensity was also tested, since the LED technology can produce high light intensity without heat damage to plants. The objectives of this study were to compare the efficiency of LED light sources with fluorescent white light in experimental wheat cultivation and to reveal how the light quality and quantity affect wheat growth and development, metabolic processes of flag leaves, grain yield and flour quality.

## Materials and Methods

### Plant Materials and Growth Conditions

A facultative genotype of hexaploid wheat (*T. aestivum* ssp. *aestivum* cv. ‘Mv Kikelet’) was obtained from the Martonvásár Cereal Gene Bank (Agricultural Institute, Centre for Agricultural Research, Hungarian Academy of Sciences, Martonvásár, Hungary). Seeds were germinated in petri dishes between sterile filter papers soaked in MilliQ water for 3 days at 26°C. After germination plantlets with similar root lengths were placed in 4 × 4 cm Jiffy pellets (Jiffy Group, Oslo, Norway)^1^ and stored at room temperature for 2 days. The 5-day old plantlets were subjected to vernalization for 2 weeks at 4°C with a 10/14 h light/dark photoperiod and a Photosynthetic Photon Flux Density (PPFD) of 12 μmol m^-2^ s^-1^. Then the plants were grown in pots (diameter 10 cm; height 30 cm; volume 2.8 l) filled with 2:1:1 (v/v/v) mixture of garden soil, humus and sand. The pots were transferred to growth chambers (PGV-36; Conviron Env. Ltd., Winnipeg, MB, Canada), programmed with a spring-summer climatic regimen developed for winter wheat (detailed in Supplementary Table 1) for 16 weeks until maturity. During this period, the temperature and duration of illumination were gradually increased with a weekly change of program to simulate spring-summer conditions in the field. The plants were watered daily with tap water through a sprinkler. A nutrient solution containing 41 mg L^-1^ N; 7 mg L^-1^ P_2_O_5_; 21 mg L^-1^ K_2_O; 4 mg L^-1^ Mg; 5 mg L^-1^ Ca; and 1 mg L^-1^ B, Cu, Mn, Fe, and Zn was applied twice a week to ensure that neither water nor nutrients were limiting. The pots were placed randomly in the growth chambers and rearranged regularly within the treatments. Fifty plants with 45 plants m^-2^ density were grown under each light condition.

#### Light Conditions

After vernalization, plants having 3 leaves were raised under different light conditions. Six different light regimens were established using fluorescent lamps (OSRAM Lumilux T5 cool white (4000K) 54W/840 fluorescent tubes) or modules equipped with a continuous wide spectrum LED (Philips Lumileds, LXZ2-5790-y) and three narrow bands of LEDs with the dominant wavelengths of 448 nm (Philips Lumileds, LXZ1-PR01); 655 nm (Philips Lumileds, LXZ1-PA01); 750 nm (Edison Edixeon, 2ER101FX00000001). All LED modules were equipped with these LEDs and each type of LED can be independently controlled within the module. The spectral composition of light used in the experiments is composed of different combination of LEDs as presented in the Supplementary Figure 1. The most important characteristics of the light conditions are summarized in **Table 1**. The different light conditions are referred to according to their typical characteristic (except in the case of RedFR) as described in **Table 1**. The different light conditions are referred to according to their typical characteristic (except in the case of RedFR). The Fluorescence white is a white light provided by fluorescence lamps; the Pink regimen when the blue and red ratio is app. 1, similarly as in case of fluorescence lamp. The Blue regimen indicates that the blue light intensity was at least 3 times higher than the red light. In RedLL regimen, the red light is dominant, as it’s intensity is 5 times higher than the blue one. The RedHL and RedLL regimens differ mainly in the light intensity. In case of RedFR regimen, the RedLL regimen is supplemented with far-red illumination.

**Table 1:** Characteristics of light regimens.

Parameters	Light regimens					
	Fluorescent white	LED Pink	LED Blue	LED RedFR	LED RedLL	LED RedHL
PPFD (400–700 nm) (μmol m^-2^ s^-1^)	249	248	250	253	253	499
PPF (400–750 nm) (μW/cm^2^)	5855	5700	5917	5322	5252	10007
Blue (400–500 nm)	1451	2215	4402	657	653	1274
Green (500–600 nm)	2280	690	23	1020	1022	2036
Red (600–700 nm)	1841	2443	1490	3346	3437	6424
Far-red (>700 nm)	283	352	2.4	299	140	273
**Fraction (%)**						
Blue	24.8	38.8	74.1	12.3	12.4	12.7
Green	38.9	12.1	0.4	19.2	19.5	20.3
Red	31.4	42.8	25.1	62.9	65.4	64.2
Far-red	4.8	6.2	0.4	5.6	2.7	2.7
**Ratios**						
Blue: Red	0.8	0.9	3.0	0.2	0.2	0.2
Red: far-red	6.5	6.9	59.6	11	25	24
Blue: far-red	5.1	6.3	176	2.2	4.7	4.7
Energy consumption (kW)	4.69	1.24	1.42	1.09	1.02	2.09


*The Fluorescent white refers to a white light regimen provided by fluorescent lamps. The Pink regimen utilized LEDs with an equal blue and red ratio. In the Blue LED regimen, the blue light was dominant and in the RedLL and RedHL regimens red light was dominant at 250 μmol m^-*2*^ s^-*1*^ and 500 μmol m^-*2*^ s^-*1*^ light intensities, respectively. In the RedFR regimen red light was complemented with far-red light. The total Photosynthetic Photon Flux Density (PPFD) was determined with a Li-Cor quantum meter (Li-189, LI-COR, Inc., Lincoln, NE, United States). The spectral energy distribution was determined by a spectroradiometer (UBS4000, Ocean Optics Inc., United States) and the wavelength ratio was calculated with the assumption that blue light ranged between 400 and 500 nm, green light between 500–600 nm, red light between 600–700 nm and far-red between 700–750 nm bandwidth integration. The energy consumption was calculated for the whole chamber with the same illuminated area using a C.A 8230 power and quality analyzer (Chauvin Arnoux Ltd, Dewsbury, United Kingdom).*

### Methods

#### Determination of Plant Growth and Development

The developmental stages of plants were determined on at least 25 plants of each light regimen according to the Zadoks scale from Z13 to Z89 (Zadoks et al., 1974) and the plant height was measured at six time points during the light treatment: at 54, 75, 94, 114, 124, and 134 days of plant cultivation. The flowering time was also recorded.

#### Determination of Photosynthetic Activity of Leaves Under Different Light Conditions

Photosynthetic activities of plants were determined in growth chambers under each light regimen. The measurements were performed on five randomly selected fully developed flag leaves using a Ciras 2 portable photosynthesis system instrument with a narrow (2.5 cm^2^) leaf cuvette (PP Systems, Haverhill, MA, United States). The net photosynthetic rate (Pn), stomatal conductance (gs), transpiration rate (E) and intracellular CO_2_ concentration (Ci) parameters were determined at the steady-state level of photosynthesis using a CO_2_ level of 380 μL L^-1^.

Photosynthetic electron transport activity was also estimated on 15 intact attached leaves for each light regimen using a PAM-2000 fluorometer (Walz, Effeltrich, Germany). Before the measurements, the plants were dark adapted for 20 min, after which the Fv/Fm parameter (indicating the maximal quantum efficiency of Photosystem II (PS II)) was determined using a 1.0 saturation pulse (PPDF = 2000 μmol m^-2^ s^-1^). Then plants were light-adapted for 20 min and the actual quantum yield of PS II [Y(II)] was determined at the steady state level of photosynthesis. The electrontransport rate (ETR) and the non-photochemical quenching (NPQ) parameter (which reflects the heat dissipation of excess excitation energy) were also calculated at the steady state level of photosynthesis.

#### Determination of Chlorophyll and Carotenoid Contents of Flag Leaves

Chlorophyll *a* and b as well as the carotenoid contents of leaf disks were determined using a Cary-100 UV-Vis spectrophotometer (Varian, Middelburg, Netherlands) after extraction in 80% acetone, according to the method of Lichtenthaler (1987). For each measurement, 5 × 0.2 *g* leaf disks (13 mm diameter) were collected from several (randomly selected) plants. The samples were stored at -80°C until extraction.

#### Determination of Thiols in Flag Leaves

The thiols were measured from flag leaves (5 × 0.2 g) after extraction in 0.1 M HCl (1:5). The total thiol content was determined after reduction with dithiothreitol (DTT) and derivatization with monobromobimane as described in Kocsy et al. (2000). For the detection of oxidized thiols, the reduced thiols were blocked with *N*-ethylmaleimide, after which the excess of *N*-ethylmaleimide was removed with toluene (Kranner and Grill, 1996). The oxidized thiols were reduced and derivatized as described for total thiols. Thiols were analyzed after their separation by Reverse-Phase High Performance Liquid Chromatography (RP-HPLC, Waters, Milford, MA, United States) using a W474 scanning fluorescence detector (Waters, Milford, MA, United States). Amounts of reduced thiols were calculated as the differences between the amounts of total and oxidized thiols.

#### Determination of Amino Acid Content of Flag Leaves

The amount of free amino acid was determined from 300 mg samples extracted with 4 ml of 10% (v/v) trichloroacetic acid for 1 h at room temperature using a Laboshake Ls 500i instrument (Gerhardt, Germany). Then the extracts were filtered through 0.25 μm membrane filters (Nalgene, United States). Analysis of amino acids was performed using an AAA 400 amino acid analyzer (Ingos, Czechia) equipped with an Ionex Ostion LCP5020 cation-exchange column (20 cm long, 3.7 mm i.d.). Amino acids in 100 μL-aliquots were injected into the column, and separated by stepwise gradient elution at 0.30 mL/min, using a Li^+^-citric buffer system. Colorimetric detection was accomplished at 570 and 440 nm (for Pro) after post-column derivatization with ninhydrin reagent.

#### Determination of Biomass and Yield Production and of Flour Quality and Composition

At harvest, the final plant height and total biomass weight, including straw, leaves and spikes, were determined from at least 25 randomly chosen plants in each treatment. The plants were dry with 15% average moisture content. In addition, the number of spikes per plant and the grain number and weight per plant were determined. The harvest index (the percentage of grain yield in total biomass) and thousand weight of grains (TGW) were also calculated. The quality of grains was characterized from the flour milled by the use of a Chopin CD1 Laboratory mill after the seeds were conditioned to 15.5% moisture content. The flour samples were immediately cooled and stored at -20°C until compositional analysis.

Starch content of the flour was measured with a Foss Tecator 1241 instrument. The crude protein content was analyzed in duplicate with a Kjeltec 1035 Analyzer (Foss Tecator, Sweden) using the Kjeldahl method, which is consistent with International Association for Cereal Science and Technology ICC 105/2 (1995). The gluten content and gluten index (GI) were determined using a Perten Glutomatic 2200 instrument (International Association for Cereal Science and Technology ICC 137/1, 1995 and International Association for Cereal Science and Technology ICC 155, 1995). Gluten spread was measured according to the Hungarian standard MSZ 6369/5-87 (1987). This parameter provides information about the proteolytic activity of the samples by monitoring changes in the diameter of a gluten ball after 1 h at room temperature. The breadmaking quality of flour was determined by the Zeleny sedimentation test based on the sedimentation of flour in a lactic acid solution according to standard protocol International Association for Cereal Science and Technology ICC 116/1, 1995.

Size-Exclusion High Performance Liquid Chromatography (SE-HPLC) was used to determine the glutenin, gliadin and albumin+globulin contents using a modification of the Batey et al. (1991) method. Ten mg flour was suspended in 1 ml 0.5 % (w/v) sodium dodecyl sulfate (SDS) in phosphate buffer (pH 6.9) and sonicated for 15 s. After centrifugation, the supernatant was filtered on a 0.45 μm polyvinylidene fluoride (PVDF) filter. Analyses were performed on a Phenomenex BIOSEP-SEC 4000 column in acetonitrile buffer (0.05% (v/v) trifluoroacetic acid and 0.05% (v/v) acetonitrile) with a running time of 10 min (2 ml/min flow rate). Proteins were detected by absorption at 214 nm.

A modified RP-HPLC method of Marchylo et al. (1989) was used to determine the relative amounts of glutenin subunits. The gliadins were extracted with 70% (v/v) ethanol followed by 50% (v/v) 1-propanol. The glutenin polymers were then reduced with buffer [50% (v/ v) 1-propanol, 2M urea and 0.2M Tris–HCl, pH 6.6] containing 1% (w/v) DTT, and alkylated with 4-vinylpyridine. The high-molecular-mass (HMW) and low-molecular-mass (LMW) glutenin subunits were separated on a Supercosil LC-308 (300A, 3.5% carbon, 5 μm, 5 × 4.6) column.

### Statistics

The measurements and sample collection for analytical investigations were performed on flag leaves or on seeds collected from several (randomly chosen) plants in each treatment. For physiological and analytical investigations, at least 5 biological replicates were used in each measurement. Grain quality analyses were conducted in 3 biological replicates. In general, the SPSS 22 statistical program and Tukey’s *post hoc* test were used to determine differences between treatments for all investigated traits. Different letters indicate significant differences at the *P* < 0.05 level. The free amino acid concentrations were also analyzed by hierarchical clustering (distance metric: Euclidian distance, linkage method: average linking clustering) using the MultiExperiment Viewer v4.5 program.

## Results

### Plant Growth and Development Under Different Light Conditions

Effects of light composition and intensity varied with the developmental stage of the plant. In the tillering period (Z20-29), only light intensity affected the numbers of tillers: higher numbers of tillers were observed in plants grown at high light intensities (RedHL, ∼500 μmol m^-2^ s^-1^) as compared to plants grown at approximately 250 μmol m^-2^ s^-1^ light intensities (Fluorescent white, Pink, Blue, RedLL, RedFR), irrespective of light quality (**Table 2**). Later, during the stem extension period (Z30-49), both light spectral composition and intensity influenced plant elongation (jointing) and booting. Plants under the Fluorescent white, Pink and RedHL regimens had the earliest heading dates, followed by RedFR and RedLL and plants grown under the Blue regimen had the latest heading date (**Table 2**). Significant differences were found only between plants that exhibited the earliest and latest heading dates.

**Table 2:** The number of tillers, heading date and flowering time of plants grown under different light conditions.

Light regimen	No. of	Heading date^2^	Flowering time^3^
	tillers^1^	(No. of days)	(No. of days)
Fluorescent white	3.86b	86.9b	93.2b
Pink	4.08b	87.0b	94.3b
Blue	4.2b	91.9a	100.2a
RedFR	4.03b	89.2ab	97.1ab
RedLL	4.04b	89.5ab	98.6a
RedHL	5.24a	88.2b	95.3ab


*^*1*^Determined after 33 days of cultivation in Phytotron chambers. ^*2*^Age of plants when heads started to emerge from the “boot” (Z50) in 50% of the spikes. ^*3*^Age of plants when anthesis started in the 50% of the main spikes (Z60). Values are the mean ± SD of at least 25 plants per light treatment. The different letters indicate statistically significant differences at *P* < 0.05, using Tukey’s *post hoc*test.*

The latest flowering date also was recorded in plants grown under the Blue regimen -this was about 1 week later than that observed in plants grown under the Pink and Fluorescent white regimens (**Table 2**). The flowering time of plants grown under RedLL did not differ from those grown under the Blue regimen and plants grown under the RedHL and RedFR regimens did not differ significantly from any other light treatments.

**FIGURE 1:**
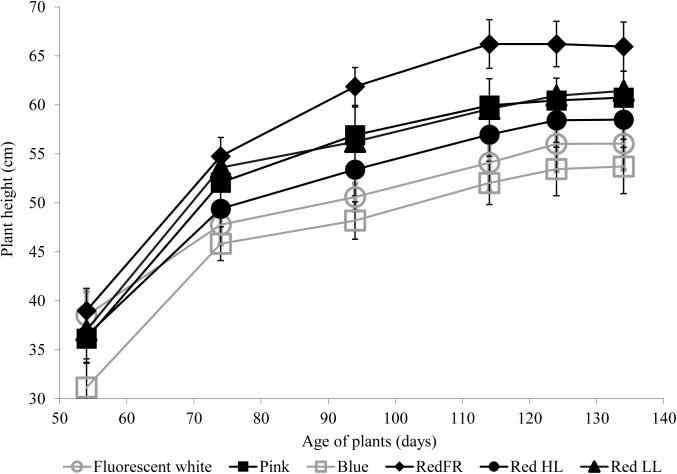
The average plant height during development under different light regimens. Values represent the mean ± SD of at least 25 plants per light treatment. The results of statistical analysis are presented in Supplementary Table 2.

The heights of plants were greatest when plants were grown under the RedFR regimen and lowest when the plants were grown under the Blue regimen (**Figure 1**). Height differences were not accompanied by changes in leaf numbers (data not shown).

During ripening (Z71-89), differences in the timing of development were not apparent under the different light sources, except under the Blue regimen, where ripening finished 4 days later than in other cases (data not shown).

### Physiological and Metabolic Response of Flag Leaves to Light Environment

#### Effect of Light Conditions on Photosynthetic Activity and Chlorophyll Content of Flag Leaves

Light quality and quantity affected the pigment content of flag leaves (**Table 3**). High contents of chlorophyll *a* and b were observed in plants grown under the Fluorescent white and RedHL regimens while significantly lower chlorophylls were found in the flag leaves of plants grown under all of the other LED light regimens. Among the low intensity LED light regimens, plants grown under the RedFR regimen had significantly lower levels of chlorophyll *a* than plants grown under the Blue or RedLL regimens while levels of chlorophyll b were not significantly different. Plants grown under the Fluorescent white and RedHL regimens also had significantly higher carotenoid contents in their flag leaves than those grown under the low intensity LED light regimens. These plants also differed in their ratios of chlorophylls to carotenoids.

**Table 3:** Chlorophyll *a* and b and carotenoid content of flag leaves grown under different light conditions.

	Chlorophyll a	Chlorophyll b	Chlorophyll a/b	Chlorophyll a + b	Carotenoid	Chlorophyll/Carotenoid
Fluorescent white	2817 ± 274a	698 ± 70a	4.04 ± 0.09a	3515 ± 356a	594 ± 80a	5.92 ± 0.40a
Pink	1890 ± 142bc	463 ± 35b	4.08 ± 0.18a	2353 ± 334bc	434 ± 36bc	5.42 ± 0.64ab
Blue	2113 ± 222b	508 ± 56b	4.16 ± 0.26a	2622 ± 277b	478 ± 59bc	5.49 ± 0.77ab
RedFR	1723 ± 173c	464 ± 57b	3.71 ± 0.19b	2187 ± 230c	414 ± 40c	5.28 ± 0.49ab
RedLL	2045 ± 91b	494 ± 16b	4.14 ± 0.36a	2539 ± 105b	492 ± 23b	5.16 ± 0.65ab
RedHL	2740 ± 70a	694 ± 31a	3.95 ± 0.10ab	3434 ± 98a	692 ± 71a	4.96 ± 0.60b


*The values are means ± SD of 5 replicates per light treatment. Different letters indicate statistically significant differences at *P* < 0.05, using Tukey’s *post hoc*test.*

**FIGURE 2:**
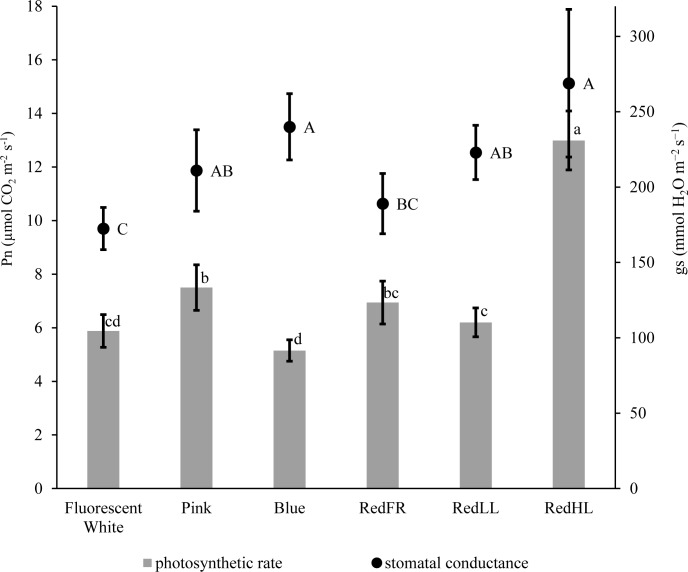
CO_2_ assimilation rate (Pn) and stomatal conductance (gs) measured on flag leaves grown under different light regimens. Values are the mean ± SD of at least 5 measurements per light treatment. The different letters indicate statistically significant differences at *P* < 0.05, using Tukey’s *post hoc* test.

CO_2_ assimilation was also influenced by light intensity and quality (**Figure 2**). The highest CO_2_ assimilation rate was produced by flag leaves of plants grown at high light intensity (RedHL). At low light intensities the photosynthetic activity of leaves was higher when the plants were grown under Pink, RedLL and RedFR regimens as compared to those grown under the Blue regimen. In spite of the low CO_2_ assimilation rate, stomatal conductance (gs) remained high when the plants were illuminated with Blue light, similar to levels observed under the RedHL. However, their values were not significantly higher than those in Pink, and RedLL regimens. Low gs values were detected when plants were grown under the Fluorescence white regimen.

Fluorescence quenching analyses revealed differences in the activity of PS II among the treatments (**Table 4**). The maximum quantum yield of PSII (Fv/Fm parameter) did not differ significantly (the average Fv/Fm was 0.785 ± 0.04), indicating that none of the growth conditions had photoinhibitory effects (data not shown). However, the actual quantum yield [Y(II), ΔF/Fm′], the electron transport rate (ETR) and heat dissipation process (NPQ) changed depending on the light quantity and quality. Not surprisingly, the lowest value of ΔF/Fm′ and the highest values of ETR and NPQ were detected on flag leaves in plants grown under the RedHL regimen. In plants grown at low light intensities, higher Y(II) and ETR and lower NPQ were detected on flag leaves illuminated with the Pink, RedLL and RedFR than by the Blue and Fluorescent white regimens.

**Table 4:** Fluorescence quenching parameters, including the actual quantum yield [Y(II)] of PS II, the non-photochemical quenching (NPQ) parameters and the electron transport rate (ETR) determined at steady state of photosynthesis of flag leaves under different light regimes.

Light regimen	Y(II)	NPQ	ETR
Fluorescent white	0.61 ± 0.016b	0.29 ± 0.013b	63.10 ± 2.764c
Pink	0.64 ± 0.027ab	0.24 ± 0.024c	73.40 ± 2.679b
Blue	0.60 ± 0.018b	0.31 ± 0.022b	65.74 ± 2.012c
RedFR	0.69 ± 0.031a	0.20 ± 0.032c	74.27 ± 2.995b
RedLL	0.68 ± 0.028a	0.22 ± 0.039c	71.72 ± 3.600bc
RedHL	0.50 ± 0.020c	0.49 ± 0.045a	94.22 ± 4.800a


*The values are means ± SD of 20 measurements per light treatment. Different letters indicate statistically significant differences at *P* < 0.05, using Tukey’s *post hoc*test.*

#### Effect of Light Conditions on Thiol Levels of Flag Leaves

**FIGURE 3:**
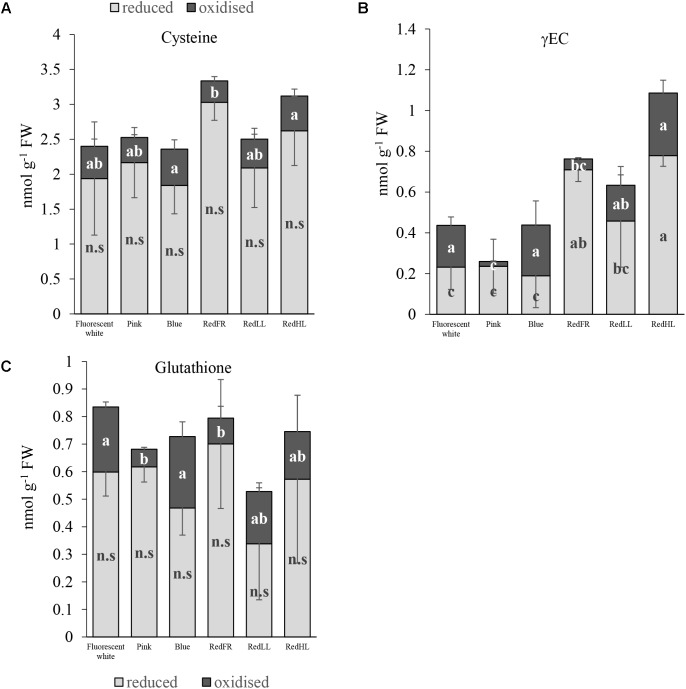
Effects of light spectral distribution and light intensity on thiol levels and forms. **(A)** Cysteine. **(B)** γ-glutamylcysteine (γEC). **(C)** Glutathione. Values are the mean ± SD of 5 biological replicates per light treatment. The different letters indicate statistically significant differences at *P* < 0.05, using Tukey’s *post hoc* test.

The light regimens affected the thiol levels, including the amounts of cysteine, the precursor of glutathione (GSH), γ-glutamylcysteine (γEC), an intermediate product in GSH synthesis, and glutathione in flag leaves. In spite of the fact that there were no significant differences in the reduced form of cysteine between the different light regimens, the total amount of cysteine was greater in plants grown under the RedFR regimen than in plants grown under Fluorescent white, Pink, Blue and RedLL regimens (**Figure 3A**). The cysteine level in leaves of plants grown under RedHL regimen did not differ significantly from any other group (**Figure 3A**). The concentration of the oxidized form of cysteine was significantly lower in RedFR regimen compared to Blue and RedHL ones. These changes resulted in an increase in the ratio of reduced and oxidized forms of cysteine in leaves of plants grown under RedFR regimen.

Among the three investigated thiols, the greatest light-dependent variation was detected for γ-glutamylcysteine (γEC) (**Figure 3B**). The highest concentration of γEC was found in flag leaves from plants grown under the RedHL regimen and the lowest in those grown under Pink regimen. In addition, the proportions of reduced and oxidized forms of γEC were affected by the light conditions. In plants subjected to Fluorescent white, Blue, RedLL and RedHL regimens, this ratio was small, while in plants produced under the Pink and RedFR regimens it was high.

The lowest level of total glutathione was found in flag leaves of plants grown under RedLL (**Figure 3C**). While the amount of reduced glutathione (GSH) was not significantly influenced by light regimens, the concentration of its oxidized form (GSSG) was significantly lower in the Pink and RedFR regimens as compared to the Fluorescent white and Blue regimens. However, the values did not differ significantly from the RedLL and RedHL regimens. The light-induced differences in oxidized glutathione contents led to significantly higher ratios of reduced and oxidized forms of glutathione (GSSG/GSH) in the Pink and RedFR regimens compared to the other ones.

#### Effect of Light Conditions on Amino Acid Contents of Flag Leaves

**FIGURE 4:**
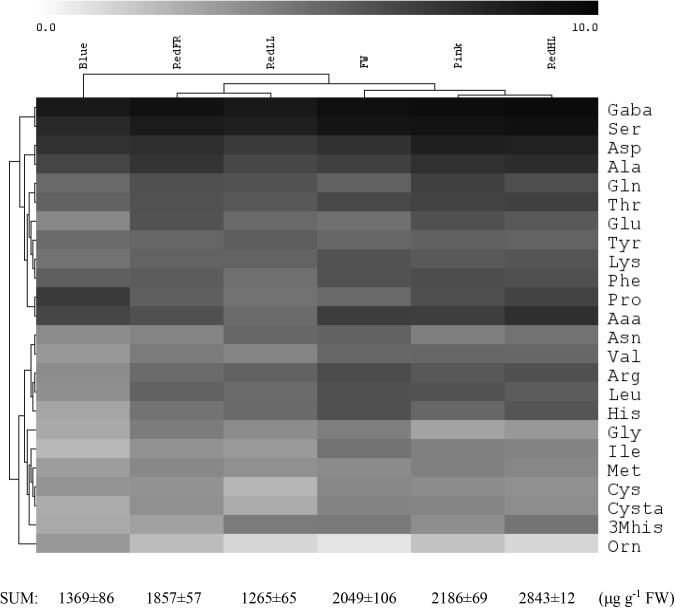
Effect of light spectrum on free amino acid levels in flag leaves. Log_2_ concentration values are shown. The total amino acid content is shown below. The values are from 5 biological replicates.

The light quality and quantity affected both the amount and the composition of the free amino acids in flag leaves (**Figure 4** and Supplementary Table 3). Low amounts of free amino acids were detected in leaves of plants grown under the Blue and RedLL regimens, while the highest amounts of free amino acids were found in plants grown under high intensity light (RedHL). GABA, Ser, Asp and Ala were the most abundant amino acids in leaves, encompassing approximately 60% of all amino acids, and their proportions were similarly affected by the various light conditions.

Based on hierarchical cluster analyses, free amino acid concentrations of flag leaves were the most different under the Blue regimen (**Figure 4**). In addition, under the Fluorescent white, Pink and RedHL treatments (grouped together) the amounts of Val, Leu, Thr, Lys, Ser and GABA in flag leaves were greater than those of the other three light regimens. Amino acids belonging to the same family were differentially affected by spectrum since they clustered into different groups. For example, in the glutamate family (about 35% of total free amino acid content is covered by this family) the concentration of Pro was high under the Blue regimen, while low values of Glu, Gln and Arg were found, but inversely, high amounts of Glu, Gln and a low amount of Pro were detected under the RedFR regimen. Only amino acids belonging to the pyruvate family (Ala, Val, Leu) were similarly affected by the same light conditions – high amounts of all three amino acids were detected in plants grown under Fluorescent white, Pink and RedHL regimens, while low amounts were found in plants grown under Blue and RedLL regimens.

### Determination of Yield Components, Flour Quality and Composition

Changes in growth, development and metabolism were manifested in the yield production and the quality of the flour (**Table 5**). The RedHL regimen had the greatest effects on biomass, grain number and grain yield when compared to plants grown under lower light intensity, irrespective of light spectra (**Table 5**). The elevated light intensity increased both the straw (biomass – grain yield) and grain production, resulting in similar harvest index as was found for the Fluorescent white regimen. Since the high light intensity enhanced tillering, the high grain yield is mostly due to elevated spike number; however, the average number of grains (32.6 ± 6.3) per spike was also the highest under this regimen.

**Table 5:** Biomass and yield production of wheat grown under different light regimens.

Light regimen	Biomass (g/plant)	Spike number per plant	Grain number per plant	Grain yield (g/plant)	TGW	Harvest-Index
Fluorescent white	8.33 ± 1.40bc	3.86 ± 0.78bc	105.2 ± 21.42cd	3.18 ± 0.62bc	30.61 ± 5.36a	38.68 ± 4.64a
Pink	8.94 ± 1.62b	4.31 ± 0.92b	118.2 ± 33.01bc	2.95 ± 0.71cd	24.27 ± 4.21b	32.41 ± 5.82b
Blue	7.77 ± 1.17c	3.64 ± 0.69c	95.9 ± 25.04d	2.56 ± 0.55d	27.08 ± 4.98b	32.69 ± 4.39b
RedFR	9.14 ± 1.43b	4.83 ± 0.80a	133.5 ± 31.48b	3.45 ± 0.81b	26.31 ± 4.58b	37.35 ± 7.91a
RedLL	8.58 ± 1.55bc	4.10 ± 0.88bc	115.1 ± 31.05c	2.73 ± 0.68cd	24.21 ± 3.91b	31.71 ± 4.61b
RedHL	12.03 ± 1.68a	4.89 ± 0.83a	159.4 ± 28.59a	4.79 ± 1.00a	30.24 ± 5.10a	39.70 ± 5.77a


*The values are means ± SD of at least 25 replicates per light treatment. Different letters indicate statistically significant differences at *P* < 0.05, using Tukey’s *post hoc* test. TGW, thousand weight of grain.*

Under low light intensities, the RedFR and Pink regimens resulted in the greatest grain number and the RedFR regimen resulted in the greatest weight. Retardation of growth in plants under the Blue regimen resulted in the lowest values of spike number, grain number and grain weight; however the difference is not always significant from other light regimens (**Table 5**). Interestingly, the plants grown under the Fluorescent white regimen produced a low number of grains with relatively high weights, resulting in a TGW that was similar to plants grown under the Red HL regimen (**Table 5**).

**FIGURE 5:**
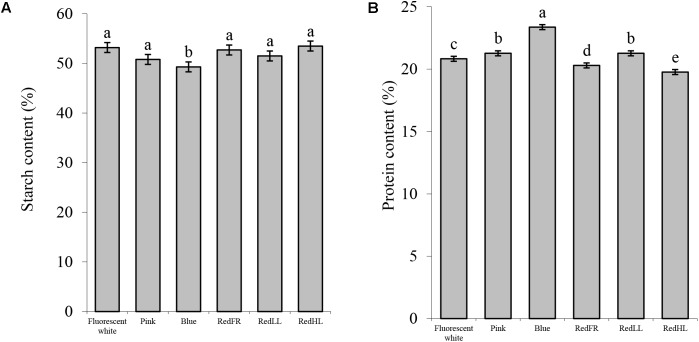
Compositional properties of wheat flour produced under different light regimens. **(A)** Starch content (%). **(B)** Protein content (%). Values are the mean ± SD of 3 biological replicates per light treatment. The different letters indicate statistically significant differences at *P* < 0.05, using Tukey’s *post hoc* test.

**FIGURE 6:**
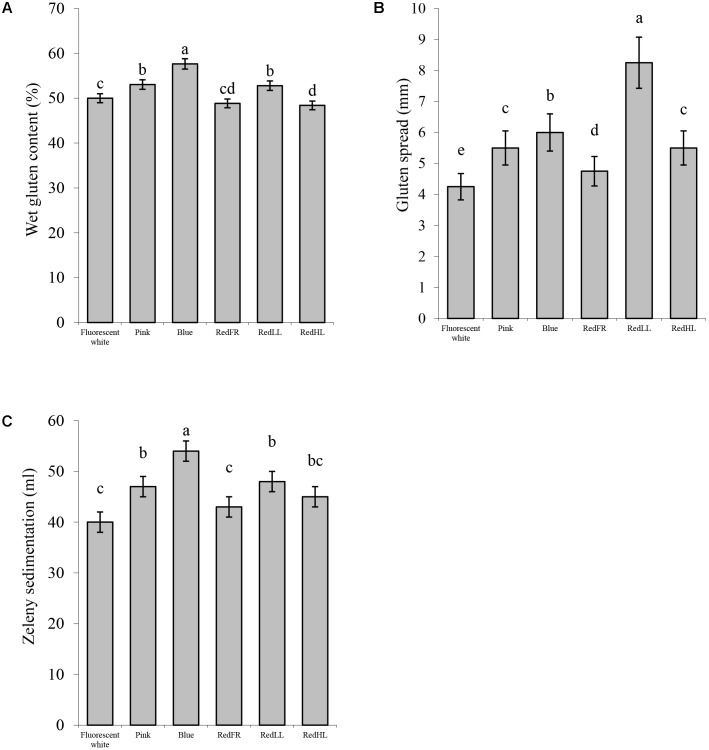
Processing quality characteristics of flour. **(A)** Wet gluten content (%). **(B)** Gluten spread (mm). **(C)** Zeleny sedimentation (ml). Values are the mean ± SD of 3 biological replicates per light treatment. The different letters indicate statistically significant differences at *P* < 0.05, using Tukey’s *post hoc* test.

Detailed analyses of compositional and processing quality traits of the grains revealed that the light quality significantly affected the composition and the quality of the flour (**Figure 5**). The starch content of flour was the lowest, while protein content was the highest in plants grown under the Blue regimen (**Figure 5**). Plants grown under the Pink and RedLL regimens also had higher protein contents than those grown under Fluorescent white regimen (**Figure 5B**). Similarly, the gluten content of the flour was highest in plants grown under the Blue regimen. Flour produced under the Pink and RedLL regimens also had higher gluten contents than that produced under the Fluorescent white regimen (**Figure 6A**).

Light-induced differences were apparent in the processing properties of the flours as characterized by the gluten index, gluten spread and Zeleny sedimentation index. The gluten index, relating to the quantity of the larger protein polymers and dough strength, was higher when the plants were grown under the RedFR regimen and lowest under the Blue regimen Supplementary Figure 2). The spread of the gluten showed considerable variation. The RedLL regimen had the largest effect on the gluten spread, significantly increasing it compared to the Fluorescent white regimen. The Blue, Pink and RedHL regimens also affected the spread of the gluten indicating greater dough extensibility/softening of these samples (**Figure 6B**). The Zeleny sedimentation provides an indication of the breadmaking quality of the flour and increased when the Zeleny sedimentations of flour from plants grown under the Blue, Pink and RedLL regimens were significantly higher than those grown under the Fluorescent white or RedFR regimens (**Figure 6C**). These results suggest that starch, protein and gluten content can be modified and breadmaking quality can be improved by an appropriate selection of light.

**FIGURE 7:**
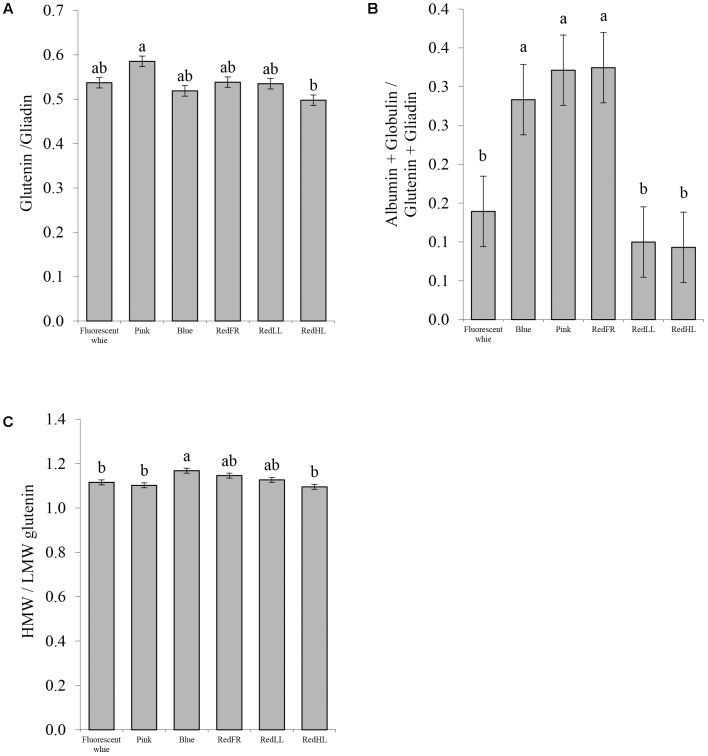
Protein composition of flour. **(A)** Ratio of the glutenins to gliadins. **(B)** Ratio of (albumin + globulin)/(glutenin + gliadin). **(C)** Ratio of high molecular weight (HMW) glutenin subunits to low molecular weight (LMW) glutenin subunits. Values are the mean ± SD of 3 biological replicates per light treatment. The different letters indicate statistically significant differences at *P* < 0.05, using Tukey’s *post hoc* test.

To understand the basis for changes in processing quality, flour protein composition was also investigated (**Figure 7**). Significant differences in the ratios of the glutenins to gliadins were found only between the Pink and RedHL regimens, with the highest value in the case of the Pink regimen. The higher value is related to greater dough strength and better breadmaking quality of the flour (**Figure 7A**). The proportions of water-soluble proteins (albumins and globulins) were significantly higher in flour from plants grown under the Pink, Blue and RedFR regimens than in those grown under other conditions (**Figure 7B**). Furthermore, the ratio of the high and low molecular weight glutenins (HMW/LMW) was higher in samples produced under the Blue regimen than those produced under the Fluorescent white, Pink and RedHL regimens (**Figure 7C**).

## Discussion

### The Efficiency of LED Lighting on Cultivation of Wheat

LED lighting offers several advantages over fluorescent lighting for controlled growth environments. First, higher light intensities can be utilized without generating excess amounts of heat. Second, LED lighting results in significantly less energy consumption, particularly when used at similar light intensities. The elevated light intensity provided by the LEDs resulted in increased biomass and yield, mainly due to the elevated photosynthetic activity of plants (**Figure 2** and **Table 5**). Indeed, increases of 40% in biomass and 60% in yield were obtained with 55% less energy consumption using LED rather than fluorescent lighting (**Tables 1, 5**). However, energy savings were even greater when LEDs were used at comparable light intensities (∼250 μmol m^-2^ s^-1^) as the fluorescent white lamps while biomass was similar or higher. These results clearly demonstrate the benefit of LEDs over conventional fluorescent white light sources.

### The Effect of Light Quality and Quantity on the Plant Growth, Development and Metabolisms

#### Growth and Development

The light environment affects morphogenetic responses of plants, including shoot branching or tillering, stem elongation and induction of flowering. These responses are mediated by several photoreceptors, such as phytochromes that are mostly sensitive to red/far-red ratios, blue receptors and photosynthetic pigments (De Salvador et al., 2008). Shoot branching is inhibited when tiller buds are shaded either by leaf extension, plant density or crop canopy (Smith and Whitelam, 1997). This phenomenon is related to increases in the proportion of far-red light and mediated by the phytochromes (Kebrom et al., 2006; Finlayson et al., 2010). Evers et al. (2006) reported that when the red/far-red ratio decreased below a certain threshold, tiller appearance ceased in spring wheat. However, the threshold depended on the light intensity. In our experiments, at early developmental stages, light intensity was the dominant factor affecting shoot branching (**Table 2**). The high number of tillers contributed to a greater production of spikes and resulted in a yield increase in plants grown under the RedHL regimen. Similarly, when effects of light intensity and spectral distribution were studied in ryegrass, the number of tillers were significantly higher in plants grown at high light intensities than those at low intensities regardless of the red/far-red ratio (Gautier et al., 1999). In these cases, the high light intensity provided enough assimilates for intense development of tillers from tiller buds and it was not necessary to prioritize resources for elongating established shoots. However, at lower light intensity the reduction in substrate availability reduced the number of tillers. We found that the spectral distribution did not cause significant differences in the number of tillers at early developmental stages of wheat (**Table 2**). It was possible that neither the plant density, nor the leaf extension shaded the plants enough to reach the threshold red/far-red ratio, even under the RedFR regimen. It seems that the light-dependent cessation of tillering is primarily determined by the light intensity rather than light spectrum until plants can produce enough assimilates for growth. However, it is important to keep in mind that other environmental factors, such as temperature, water and nutrition availability also influence the development of tillers (Assuero and Tognetti, 2010).

The spectral composition was more important than light intensity in stem elongation and resulted in greater variation in plant height (**Figure 1**). Stem elongation is regulated by the action of several photosensors in a sequential and orchestrated manner (Folta and Childers, 2008). The red and far-red light sensing phytochromes (A and B), and the blue sensing phototropins and chryptochromes contribute to the rapid inhibition of stem elongation (Parks et al., 2001), while green light antagonizes the blue- and red-induced inhibition of elongation (Folta, 2004). These effects were primarily studied on hypocotyl elongation during the transition from darkness to light (Appelgren, 1991). Consistent with these results, the RedFR regimen promoted stem elongation, while the Blue regimen inhibited stem elongation resulting in plants that were either the tallest or shortest in plant height (**Figure 1**). With the RedFR treatment, only the internodes were elongated and there was no difference in the number of internodes (data not shown), similar to the findings of (Kim S.J. et al., 2004). Interestingly, the Pink, RedLL and RedHL regimens resulted in plants of similar heights, indicating that the light intensity and red light play marginal roles in stem elongation. It should be mentioned, however, that the spectrum of the Blue regimen consisted of blue and red light between 400–500 nm and 600–700 nm, respectively; while the intermediate region (between 500 and 600 nm) was absent (Supplementary Figure 1 and **Table 1**). In other radiation regimens, including the Fluorescent white regimen, the spectra also contained the green region. These results confirm that the low fluence of blue light and high proportion of far-red light activate the typical shade avoidance syndrome during stem elongation. The antagonist effect of green light on growth inhibition was also observed. Light quality, quantity and duration control flowering through the interaction of numerous photoreceptors including phytochromes, chryptochormes and photosynthetic pigments. Some of them operate antagonistically as the detailed mechanisms are discussed by (Lin, 2000; Thomas, 2006). In this way, blue, red and far-red light affect flowering. In our experiments, we found that both heading date and flowering time were delayed when plants were grown under the Blue regimen (**Table 2**), which may be due to a decrease in assimilate production. High light intensity did not accelerate the flowering time, suggesting that light quantity plays a lesser role in complex flowering signals. The shortest flowering times were detected in plants grown under the Fluorescent white and Pink regimens, where the blue and red ratio was around one, however they did not differ significantly from RedFR and RedHL regimens (**Table 2**). It is possible that the relative proportion of blue and red may also affect the flowering time.

#### Pigment Composition and Photosynthetic Activity of Plants

Photosynthetic pigments absorb and convert the light energy into chemical energy via complex photosynthetic machinery. Blue and red irradiations play an active role in photosynthesis and also stimulate chlorophyll and carotenoid biosynthesis (Fan et al., 2013). In the present study, light intensity caused the largest effects on photosynthetic processes, which were manifested in elevated CO_2_ assimilation capacities, decreases in the conversion ratio of absorbed light energy to photochemistry in PS II, as indicated by the low Y(II) value, and increases in heat dissipation as detected by the high NPQ. However, spectral composition also modified both the CO_2_ assimilation and the electron transport processes at low light intensity. A relatively high photosynthetic activity (Pn) was achieved with high red irradiation contribution (at least 50%), without over-excitation of PS II, as the high Y(II) and low NPQ parameters indicated, especially when far-red irradiation was applied (**Table 4**). Similar results were found by (Zhen and van Iersel, 2017). In contrast to red light application, low Pn and high NPQ values were detected when blue light was dominant. These data indicate that the high proportion of blue irradiation (above 70%) is not the most efficient for either CO_2_ assimilation (in spite of the fact that the stomata are opened), or photosynthetic electron transport processes. Blue light may overexcite PSII reaction centers resulting in lower photochemical utilization of absorbed light energy (low Y(II)) and higher heat dissipation of excess light energy (high NPQ), similar to what is observed under high light intensity. A similar conclusion was obtained by Hogewoning et al. (2010), when the effects of blue irradiation were studied on cucumber plants grown under different combinations (0-100%) of red and blue light. In other studies, the high proportion of blue light also caused a decrease in CO_2_ assimilate (Kim H.H. et al., 2004; Hogewoning et al., 2010).

It has long been known that bright, blue and red lights induce stomatal opening (Assmann, 1993); however, blue light is more efficient than red (Kim H.H. et al., 2004). When different Arabidopsis mutant plants were used, Talbott et al. (2003) found that the blue light-specific stomatal opening could be reversed either by green light or by far-red light, which was mediated by phytochromes. We also found that the RedHL and Blue regimens induced stomatal opening, while the stomata were more closed under the RedFR and Fluorescent white regimens. It seems that neither the red/far-red, nor the blue/red ratio correlated to stomatal movements. Rather the low blue/far-red ratio, as calculated 2.17 for Fluorescent white and 2.77 for RedFR regimens, induced a slight stomatal closure (**Table 1** and **Figure 2**).

Interestingly, less photosynthetic pigments, chlorophylls (*a*+b) and carotenoids were synthesized when LEDs were used for illumination as compared to fluorescent white light, with the exception for RedHL. This phenomenon was also observed by Wang (2015). Similarly, the lowest chlorophyll (*a* and b) content were obtained under RedFR regimen as also found by Li and Kubota (2009), however, it was not different significantly from Pink regimen. The relative proportion of chlorophyll b was higher under the RedFR regimen, while the proportion of carotenoids increased in plants grown under the RedHL as compared to other LED regimens. These changes may correlate to phytochrome (PFr) mediated inhibition of chlorophyll biosynthesis as found by Inagaki et al. (2015) or to a light–induced accumulation of carotenoids to protect the photosynthetic apparatus under bright light (Steiger et al., 1999).

#### Changes in Redox Homeostasis and Free Amino Acid Content Under Different Artificial Radiation

Glutathione is a central component in regulation and maintenance of cellular thiol redox homeostasis and plays important roles in plant development (Cejudo et al., 2014). Although the pool sizes of glutathione were slightly, but not significantly affected by the various light regimens in our experiment, the ratios of reduced to oxidized forms were significantly higher in Pink and RedFR regimens than in other regimens. The high proportion of reduced glutathione ensures the effective removal of excess H_2_O_2_, which is one of the reactive oxygen species (Foyer and Noctor, 2011). Although plants grown under RedHL exhibited great formation of γEC which is the rate limiting step in GSH synthesis, a simultaneous great GSH accumulation was not observed, indicating its further metabolism. It seemed that among the artificial radiation tested, the far-red illumination affected mainly the ratio of reduced to oxidized forms of glutathione and γ-glutamylcysteine. These results also proved that the spectral conditions affect the GSH/GSSG ratio. Its maintenance is very important for the control of the cellular redox environment affecting many redox-dependent metabolic processes such as the Calvin cycle, starch metabolism and lipid synthesis, as indicated by Geigenberger and Fernie (2014). A direct involvement of GSH in the control of physiological processes by light conditions is possible through a glutathione S-transferase interacting with the far-red insensitive 219 protein since the expression of the gene encoding this glutathione S-transferase proved to be differentially regulated by far-red light and other spectral components (Chen et al., 2007). In the light regulation of metabolic processes, GSH is interconnected with other redox compounds such as thioredoxins and glutaredoxins through ferredoxins and NADPH and involved in photosynthetic electron transport (Dietz and Pfannschmidt, 2011). Both past and present studies suggest that alterations in red/far-red ratios are very important in the control of these processes.

Nitrate-assimilation and incorporation of N into amino acids need both reducing capacity and energy derived from photosynthesis, therefore the effect of light conditions on metabolism can be monitored by measuring free amino acid levels. Their availability is important for the synthesis of proteins and several other metabolites. The greater photosynthetic activity that was induced by the higher light intensity of the RedHL regimen was accompanied by a greater total free amino acid content. At low intensity, the high red/far-red ratios of the Blue and RedLL regimens were associated with lower total free amino acid contents compared to other spectral conditions. These results indicate that the amount and the availability of free amino acids for the synthesis of proteins and other metabolites depend on the quantity and quality of radiation. Interestingly, within biosynthetic families, the amounts of amino acids were affected differently by the light conditions. This also indicated the importance of the spectral conditions in the adjustment of the levels of the individual free amino acids. Their amounts can also regulate the levels of several metabolites synthesized from individual amino acids. Glu has a central role in metabolism as a precursor of GSH, Pro and polyamines through Arg. Accordingly, the greater Pro content was accompanied by lower Glu content in plants grown under the Blue regimen compared to the other spectral regimens except for Pro in RedHL and Glu in Fluorescent white regimens. The Pro level was greatly affected by light regimens as shown by its fourfold greater concentration in plants grown under the Blue regimen rather than the RedFR regimen. A similar large light-induced variation in Glu content was observed indicating a possible coordination of the metabolism of these two amino acids. The greatest Pro content in wheat was observed under the Blue regimen. Blue irradiation also increased Pro content in tomato (Kim et al., 2013). Other evidence for the regulatory role of light spectrum on Pro was found in Arabidopsis in which red light sensing phytochromes were found to control the Pro content (Yang et al., 2016). The light-dependent adjustment of Pro content during growth and development under various environmental conditions could be very important since it is a multifunctional amino acid participating in the control of signaling, energy status, osmotic and redox conditions (Szabados and Savouré, 2010).

### The Effect of Light Quality and Quantity on the Flour Quality and Composition

Previous studies showed that low light intensity can affect starch granules resulting in a decline in the grain yield and starch content of the kernels (Li et al., 2008). Furthermore, B-type starch granules (<9.9 μm) proved to be much more sensitive to shading during the grain filling period than A-type granules (>9.9 μm) (Li et al., 2008). Dong et al. (2014) showed that the soluble sugar content of the grain and the accumulation of carbohydrate decreased when narrow spectra such as red or red and blue lights were used. The accumulation of starch under the blue light was less in comparison with that in the red light. The same authors also stated that white light can provide good quality and high yield.

In this study it was found that the Blue and Pink regimens had the greatest effect on flour quality. The starch content of flour decreased probably due to the reduced CO_2_ assimilation capacity of leaves in plants grown under Blue regimen as compared to other light regimens, in agreement with previous studies (**Figure 5A**), (Dong et al., 2014). Protein accumulation was not inhibited so much when the plants were grown under the Blue regimen, so the protein and gluten content and the Zeleny sedimentation increased (**Figures 5B, 6**). Furthermore, Pink and RedLL regimens also increased the protein and gluten content, gluten spread and Zeleny sedimentation as compared to the Fluorescent white regimen. Interestingly RedLL had the greatest effect on gluten spread. Gluten proteins have crucial importance in breadmaking performance of wheat flour as they determine dough strength and elasticity by forming a protein network. The quality of the gluten protein network affects the strength and extensibility of the dough, which determines its gas holding capacity and therefore defines bread volume. The more extensible dough has higher gluten spread, which also provides information about the proteolytic activity of the dough. Baking quality is characterized by the Zeleny sedimentation that shows how the flour sediments in a lactic acid solution after hydration and swelling of flour components. As demonstrated by the Zeleny sedimentation, the Blue, Pink and RedLL regimens resulted in an improvement in breadmaking quality from milling (>35 ml) to premium (>45 ml) quality of flour (**Figure 6C**).

Protein composition was also affected by light (**Figure 7**). The ratio of the HMW and LMW glutenins and the ratio of the soluble proteins related to insoluble proteins (Alb + Glob/Glu + Gli) increased in plants grown under the Blue regimen, while the Glu/Gli ratio increased under the Pink regimen, indicating that there were positive changes in the protein composition and processing quality of wheat. Although both the strength and the extensibility of the dough increased as suggested by the HMW/LMW, Glu/Gli and the gluten spread values, they ultimately contributed to the improvement of breadmaking quality, as indicated by the Zeleny sedimentation. The RedLL regimen moderately influenced the composition and quality traits of the grain by increasing protein and gluten content, gluten spread and the Zeleny sedimentation as compared to Fluorescent white regimen. Interestingly, in spite of the fact that the high light intensity provided by LEDs with RedHL regimen resulted in elevated biomass and yield production, the RedHL regimen caused the smallest effect on the studied compositional and processing quality traits compared to grain of plants grown under Fluorescent white light. These results indicated that the light quality may also be an important factor determining grain composition.

## Conclusion

The decreasing price of LEDs allows the modernization of plant growth chambers with different LED light sources. This paper demonstrated the efficiency of LED technology in wheat cultivation under artificial light conditions and also specified the dominant light factors affecting wheat development and metabolism. In our experiments, the light intensity primarily determined the photosynthetic activity and yield production of plants and was the main factor during the tillering phase. Light quality affected stem elongation, which was influenced by blue, green and far-red light antagonistically. The flowering time was delayed when the Blue regimen was applied, while the shortest flowering time was achieved when the blue and red ratio was around one. Light quality and quantity also changed the metabolism of leaves. In spite of the fact that the Blue regimen stimulated stomatal opening, the CO_2_ assimilation capacity of flag leaves remained low. The redox states of thiols were affected mainly by the RedFR regimen through modifying the ratio of reduced and oxidized forms of redox sensitive metabolites, such as γ-EC, cysteine and glutathione. The Blue regimen stimulated proline metabolism. Application of LEDs, especially when Blue, Pink and RedLL regimens were applied, improved the milling quality of flour via modification of strength and the extensibility of the dough and solubility of proteins including the gluten content. Taken together, these results demonstrate that the utilization of LED lighting technology is desirable for indoor plant cultivation. The modification of radiation conditions during development makes it possible to optimize growth conditions for wheat and to obtain desired traits and products.

## Author Contributions

IM, MA, and ÉD developed plant materials. MH and NH controlled the light conditions. IM, GK, MR, GS, MP, DT, LS-S, SA, and ÉD carried out experimentation and data evaluation. GG, ÉD, and GK established background for research. ÉD, GK, MR, IM, SA, and GG wrote the paper.

## Conflict of Interest Statement

The authors declare that the research was conducted in the absence of any commercial or financial relationships that could be construed as a potential conflict of interest.

## Footnotes

1 http://jiffypot.com
