# Supplementary figures and images for: LED Lighting – Modification of Growth, Metabolism, Yield and Flour Composition in Wheat by Spectral Quality and Intensity
Source: Front Plant Sci. 2018 May 4;9:605. doi: 10.3389/fpls.2018.00605 (PMC5945875; doi:10.3389/fpls.2018.00605)

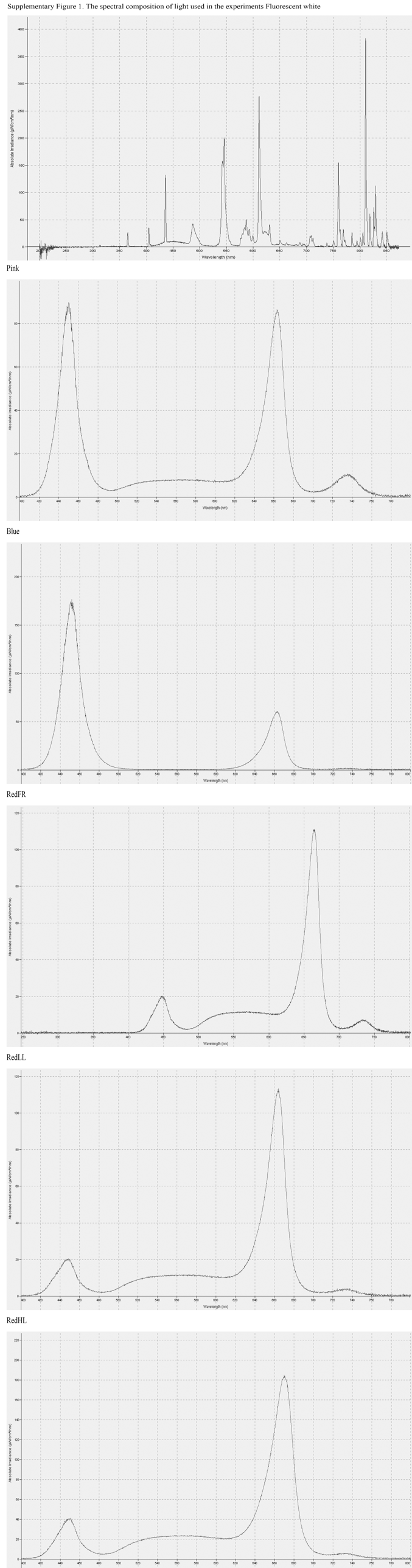

Supplement: Supplementary file 1 [file Image_1.TIF]

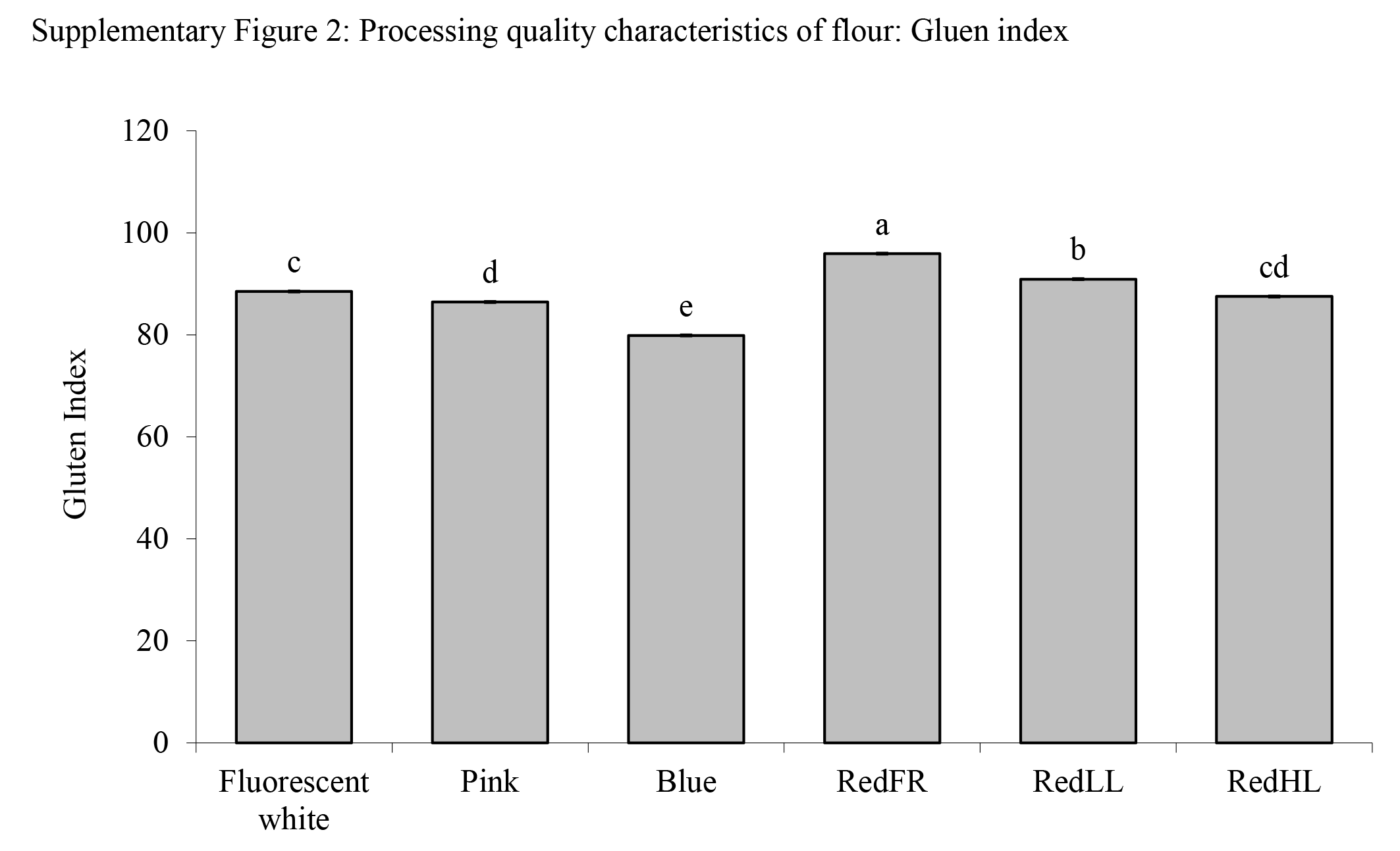

Supplement: Supplementary file 2 [file Image_2.TIF]
