# Supplementary material for: LED Lighting – Modification of Growth, Metabolism, Yield and Flour Composition in Wheat by Spectral Quality and Intensity
Source: Front Plant Sci. 2018 May 4;9:605. doi: 10.3389/fpls.2018.00605 (PMC5945875; doi:10.3389/fpls.2018.00605)
Supplement: Supplementary file 3 [file Table_1.PDF]

Supplementary Table 1. Details of spring-summer climatic program for winter wheat cultivation

	Temperature (°C)		Duration of illumination (hours:minutes)
	night	day	
1. week	5	12	12
2. week	6.5	15	13
3. week	8	16	14
4. week	9	18	14:30
5. week	10.5	19	15
6. week	11.5	20.5	15:30
7. week	12	21	15:30
8. week	13.5	22.5	15:45
9. week	14	23	15:45
10. week	14.5	24	15:45
11. week	15	24.5	15:45
12. week	16	25.5	15:45
13. week	16.5	26	15:30
14. week	17	26.5	15:30
15. week	17	27	15:30
16. week	17	27	15:30
