# Supplementary material for: LED Lighting – Modification of Growth, Metabolism, Yield and Flour Composition in Wheat by Spectral Quality and Intensity
Source: Front Plant Sci. 2018 May 4;9:605. doi: 10.3389/fpls.2018.00605 (PMC5945875; doi:10.3389/fpls.2018.00605)
Supplement: Supplementary file 4 [file Table_2.PDF]

Supplementary Table 2. The average plant height during the development under different light conditions.

Age of plants (day)	Plant height (cm)					
	Fluorescent white	Pink	Blue	RedFR	RedLL	RedHL
54	38.50a	36.16b	31.16c	39.00a	37.00ab	36.44b
74	47.77c	52.08b	45.84d	54.76a	53.60ab	49.40c
94	50.60cd	56.92b	48.20d	61.88a	56.24b	53.40bc
114	54.10cd	59.95b	52.04d	66.22a	59.60b	56.98bc
124	56.03cd	60.47b	53.46d	66.22a	60.94b	58.44bc
134	56.05cd	60.75bc	53.72d	65.96a	61.44b	58.50c

Values are the mean of at least 25 measurements per light treatment. The different letters indicate statistically significant differences at $P < 0.05$, using Tukey’s *post hoc* test.
