# Supplementary material for: LED Lighting – Modification of Growth, Metabolism, Yield and Flour Composition in Wheat by Spectral Quality and Intensity
Source: Front Plant Sci. 2018 May 4;9:605. doi: 10.3389/fpls.2018.00605 (PMC5945875; doi:10.3389/fpls.2018.00605)
Supplement: Supplementary file 5 [file Table_3.PDF]

Supplementary Table 3. The light quality and quantity effect on the amount of the free amino acids in flag leaves

	Fluorescent	Red HL	Red LL	Pink	Blue	Far-red
3Mhis	20.79±5.32ab	24.49±0.82a	20.02±5.39ab	12.66±1.12bc	6.35±0.92c	8.06±2.25c
Aaa	116.05±18.94b	181.59±23.19a	30.39±8.34d	111.16±7.65b	86.74±3.41bc	65.30±24.51cd
Ala	104.67±6.75c	205.16±18.24a	86.01±11.70c	172.22±8.59b	87.55±3.14c	152.72±7.21b
Arg	71.49±17.11a	64.21±9.00a	38.58±6.94b	50.66±2.39ab	13.55±0.53c	30.71±0.43bc
Asn	39.47±6.27a	25.67±2.54abc	33.96±11.21ab	19.00±6.11bc	13.36±2.08c	16.14±5.59c
Asp	168.52±8.83bc	294.80±20.20a	124.46±8.24c	312.70±12.71a	164.03±35.47bc	179.69±16.43b
Cys	15.50±0.77a	11.94±4.63ab	5.06±0.47b	13.26±0.88ab	11.17±4.42ab	11.43±3.61ab
Cysta	16.49±6.28a	12.77±4.22a	6.26±0.42a	15.81±6.05a	6.23±0.99a	12.13±2.11a
Gaba	623.23±14.47b	766.46±28.25a	385.43±22.81d	712.55±35.64a	400.19±40.39d	532.46±29.97c
Gln	38.78±0.62bc	68.66±19.11b	59.28±9.29bc	105.41±4.50a	32.01±3.44c	64.54±16.87b
Glu	28.00±3.58bc	49.95±5.27a	32.77±3.00b	65.20±4.70a	15.45±0.79c	60.78±12.41a
Gly	19.27±0.62a	9.93±1.35bc	13.11±2.05b	7.74±0.87c	6.87±1.81c	19.67±2.88a
His	65.00±9.26a	54.63±19.62ab	30.94±4.95bcd	35.95±1.85bc	7.29±2.23d	25.07±1.42cd
Ile	26.80±5.13a	17.23±2.70b	9.60±1.21cd	18.06±1.80b	4.61±0.10d	11.71±0.94bc
Leu	64.61±2.33a	46.98±1.99b	30.17±1.82d	59.96±2.53b	12.24±2.68e	39.32±3.72c
Lys	61.50±4.04a	54.82±0.89ab	37.93±4.44c	49.64±1.37b	25.23±6.08d	38.14±1.72c
Met	13.61±1.83ab	15.14±3.16ab	12.13±2.25ab	18.12±4.06a	8.70±0.20b	14.77±3.75ab
Orn	1.81±0.16c	2.42±0.59bc	2.41±0.72bc	3.72±1.65bc	9.98±0.70a	4.32±0.61b
Phe	62.46±2.75ab	66.49±3.53ab	27.73±8.47c	69.72±4.33a	42.17±3.34bc	45.47±18.79abc
Pro	33.12±6.81d	93.65±22.77ab	26.63±7.92d	68.63±2.61bc	123.81±12.94a	41.96±10.40cd
Ser	471.53±18.08b	602.23±47.30a	316.38±24.42c	501.34±41.66b	212.64±3.93d	364.35±11.35c
Thr	80.36±9.08b	102.71±4.27a	51.86±4.85cd	98.00±6.54a	38.44±2.38d	63.71±6.55c
Thy	34.88±7.58a	38.07±8.19a	44.70±10.77a	40.63±4.48a	30.71±2.68a	35.018±5.05a
Val	33.61±3.34a	33.37±4.36a	16.31±1.51b	34.91±1.43a	10.09±1.11c	20.38±0.96b
